# Incidental Cutaneous Reaction Patterns: Epidermolytic Hyperkeratosis, Acantholytic Dyskeratosis, and Hailey-Hailey-Like Acantholysis: A Potential Marker of Premalignant Skin Change

**DOI:** 10.1155/2011/645743

**Published:** 2011-03-30

**Authors:** Erich M. Gaertner

**Affiliations:** SaraPath Diagnostics, 2001 Webber Street, Sarasota, FL 34239, USA

## Abstract

Focal acantholytic dyskeratosis (FAD), epidermolytic hyperkeratosis (EHK), and Hailey-Hailey-like acantholysis (HH) represent unique histology reaction patterns, which can be associated with defined phenotypic and genotypic alterations. Incidental microscopic foci demonstrating these patterns have been identified in skin and mucosal specimens in association with a gamut of disease processes. These changes, when secondary, are of unclear etiology and significance. The following study further analyzes the incidence and association of these histologic patterns in a routine pathology/dermatopathology practice.

## 1. Introduction

A variety of incidental microscopic cutaneous changes have been described in skin and mucosal specimens. Whether these represent spurious changes of no consequence, or true manifestations of underlying cellular alterations, remains unclear. Incidental FAD, HH, and EHK have been reported in association with a wide variety of benign and malignant skin conditions. (Table 2) Some authors believe these changes represent markers for underlying widespread cellular damage, likely from prolonged sun/ultraviolet light exposure. Several studies show an association of these changes with preneoplastic lesions and malignancy, supporting this theory. However, others cite a variety of clinical and pathologic evidence to refute this. A potential association between EHK, and possibly FAD, with atypical/dysplastic nevus has also been reported, although not uniformly.

## 2. Material and Methods

247 consecutive skin specimens covering a three-month period (1/04-3/04) were reviewed by the author to identify incidental foci of Hailey-Hailey-like acantholysis (HH) and focal acantholytic dyskeratosis (FAD). Subsequently, 500 consecutive skin specimens were reviewed by the author (8/08-9/08) at a different institution to evaluate for incidental foci of epidermolytic hyperkeratosis (EHK). An incidental focus was defined as a minor histologic finding occurring within a biopsy or excision specimen demonstrating a separate, primary process. All cases in which these patterns comprised the primary process were excluded. HH, FAD, and EHK patterns were defined utilizing standard diagnostic criteria. (Table 1) All cases were formalin fixed, paraffin embedded, and hematoxylin and eosin stained as per standard protocol.

## 3. Results

Six cases of incidental FAD and HH were identified in the 247 skin specimens reviewed, representing 2.4% of the total reviewed. Of the six cases, three were shave biopsies (chest, back, and face), two were excisions (back, face), and one was a punch biopsy (scalp). Three specimens had an HH-like pattern (1.2% of total), and three had an FAD pattern. Four patients were male and two were female. The average patient age was 68 years (range 41–86). Three cases were associated with malignant or premalignant epidermal neoplasia: basal cell carcinoma (HH), actinic keratosis (HH), and melanoma in situ (FAD) (Figure 3). Two were associated with significant inflammation: inflamed seborrheic keratosis (HH) and bullous lichen planus (FAD) (Figure 4). One biopsy was of lichen simplex chronicus (FAD) (Figure 2). The average diameter of acantholysis was 0.3 mm, with a range of 0.1 mm to 0.5 mm. Four cases were associated with prominent solar elastosis.

**Figure 1:**
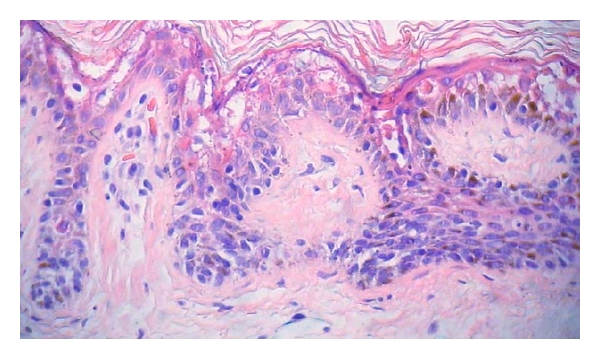
Incidental epidermolytic hyperkeratosis occurring in association with a dysplastic junctional nevus. (200X magnification, hematoxylin, and eosin stain).

**Figure 2:**
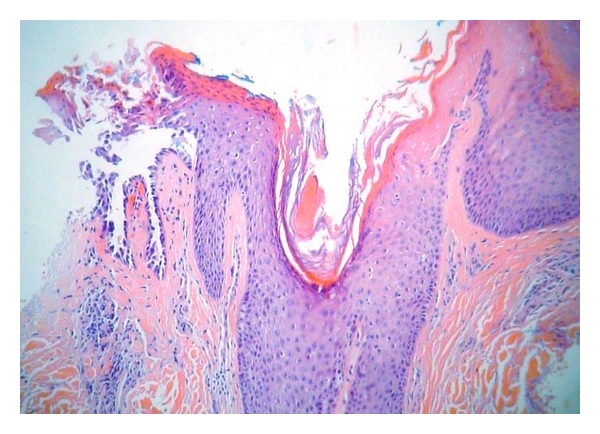
Incidental focal acantholytic dyskeratosis occurring in a biopsy of lichen simplex chronicus. (200X magnification, hematoxylin, and eosin stain).

**Figure 3:**
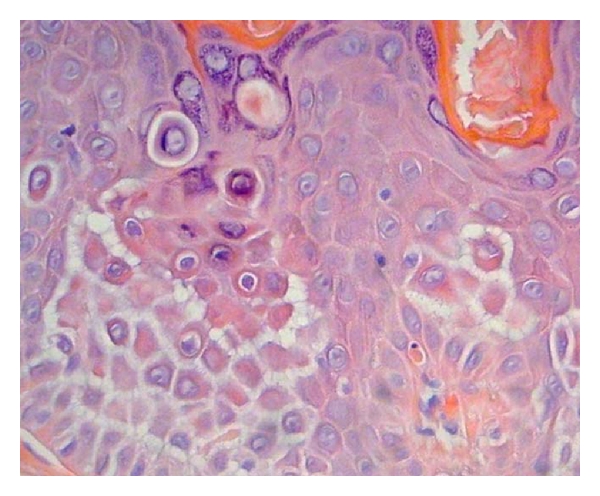
Incidental focal acantholytic dyskeratosis associated with prominent solar elastosis. This occurred in association with an excision of a melanoma in situ. (400X magnification, hematoxylin, and eosin stain).

**Figure 4:**
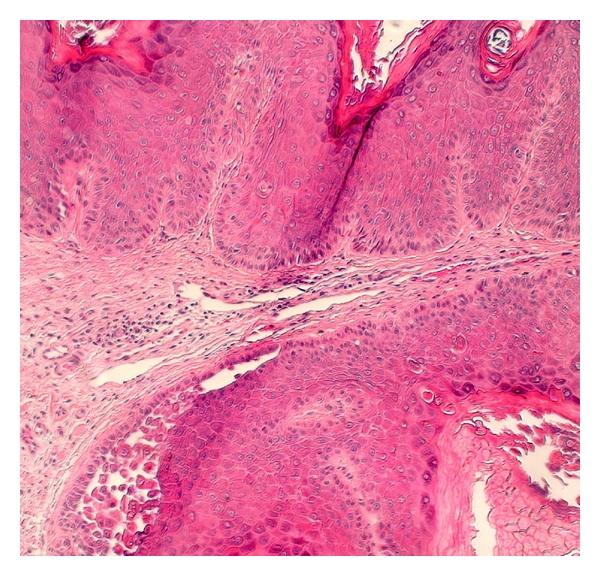
Incidental focus of Hailey-Hailey-like acantholysis, occurring in association with a benign keratosis with features of seborrheic keratosis. (200X magnification, hematoxylin, and eosin stain).

Nine cases of EHK were identified in the 500 skin specimens reviewed, representing 1.8% of total. Of the nine cases, six were excisions (arm, cheek, back [9], and neck [9]), and three were shave biopsies (thigh and back [9]). Four patients were male and five were female. The average patient age was 68 years (range 54–85). Six cases were associated with excisions of epidermal malignancies: basal cell carcinoma [16], squamous cell carcinoma [9], and melanoma in situ [7]. Three cases were associated with biopsies of dysplastic/Clark's nevi (out of a total of 82 dysplastic nevi diagnosed during this period) (Figure 1). The average diameter of EHK was 0.15 mm, with a range of 0.05 mm to 0.8 mm. Six cases were associated with prominent solar elastosis. None were associated with significant inflammation.

## 4. Discussion

Several previous studies have reviewed incidental foci of FAD, HH, or EHK. In the largest studies, incidental acantholysis was identified in 14 of 9000 specimens [35], and incidental FAD was identified in 8 of 5800 skin specimens [21]. Incidental EHK was identified in 21 out of approximately 30,000 specimens [1] and 41 out of 21,176 consecutive specimens [34]. In another study, these incidental reaction patterns were identified in 2.6% of the 1606 reviewed skin specimens, with incidental FAD identified in 0.44% (7 cases), EHK in 1.2% (19 cases), and HH in 0.68% (11 cases) [20]. The reported age of affected patients ranged from 3 to 87 years, with the largest study yielding a mean age of 55 years for FAD and 45 years for EHK. (8,25) There is no reported significant sex difference for incidental FAD or HH patterns, but incidental EHK was twice as common in men than women in the largest study [1]. 

These foci occur in both sun-exposed and sun-protected areas, with occurrence on the trunk more common than the head/neck or extremities. The involved areas are generally quite small, often involving only a single rete ridge, although have been reported as large as 12 mm [21]. They generally occurred in clinically normal skin adjacent to the primary lesion although they can occur within the lesion. Occasionally, these patterns are combined in a single specimen, and foci of acantholysis with overlapping histologic patterns have been described. Other reported incidental acantholytic patterns include those with features of pemphigus vulgaris and superficial pemphigus [35]

The etiology of these changes is unclear. Incidental FAD, HH, and EHK have been previously associated with premalignant and malignant lesions. This was also noted in the current study, with incidental FAD and HH showing a 50% association (3 of 6 cases), and EHK showing a 100% association (9 of 9 cases if one includes dysplastic nevus in this category). Of note, 3 of 9 cases of incidental EHK were associated with dysplastic nevus. A total of 82 dysplastic nevi were diagnosed during the study, yielding a low sensitivity of 3.7% for EHK as a marker for dysplastic nevi, although of a high specificity, given no cases were identified in ordinary nevi. 

Incidental EHK has been reported to be a useful marker when present for dysplastic nevus, being found more commonly in or around nevi with architectural disorder than in common melanocytic nevi [5]. This was confirmed in a follow-up study [6], although the association was of limited utility, given a low sensitivity, with only 4% of atypical nevi showing incidental EHK [5]. A follow-up study however failed to identify this association but did identify one between incidental FAD and dysplastic nevi [4]. 

Given the frequent association of incidental FAD, HH, and EHK with neoplastic or preneoplastic lesions, some authors postulate these changes are markers of widespread mutagenic change in the skin secondary to prolonged UV exposure “field cancerization” [20]. Ultraviolet light exposure has been linked to other known acantholytic disorders, such as transient acantholytic dermatosis, and has been reported to affect intercellular adhesion molecules. In the current cases, the majority were associated with prominent solar elastosis, reflecting longstanding solar damage. However, most studies show no statistically increased incidence in sun-exposed versus non-sun-exposed skin for these incidental changes. Additionally, these foci occur in clinically and microscopically normal skin and mucosa, and one published abstract-postulated incidental EHK may be a common subclinical finding, with more than 20 microscopic foci present in an individual's normal skin [34]. 

Significant inflammation was also identified in several current cases, and multiple published case reports associate a variety of inflammatory dermatoses with incidental acantholysis. As with solar damage, inflammation can affect intercellular adhesion in a variety of ways, to include changes in intracellular adhesion molecules and cytokine release. The defined genetic abnormalities described for HH and EHK as primary processes have not been identified in these incidental foci although they have not been well studied. As in previous studies, the foci of reported incidental FAD, HH, and EHK were quite small (0.3 mm for FAD/HH and 0.15 for EHK). Most of them were found in uninvolved skin adjacent to the primary lesion, but some were found within the lesion proper. Several of the primary disease processes in the current series have not been previously reported in association with incidental acantholysis (FAD and HH), such as bullous lichen planus.

Incidental FAD, HH, and EHK are interesting, uncommon cutaneous changes of unclear etiology and significance. In the current paper, these foci were associated with epidermal neoplasia, solar change, and inflammation. An association with premalignant change and malignancy has been previously reported, although not uniformly. An association between dysplastic nevi and incidental EHK and FAD has also been noted and may be of limited diagnostic utility. The exact etiology of these secondary patterns is unclear, but their presence may reflect more widespread, subclinical cutaneous injury.

##  Statement of Financial Interest

No competing financial interest exists. The research received no specific grant from a funding agency in the public, commercial, or not-for-profit sectors.

## Figures and Tables

**Table 1:** 

Diagnosis	Histology
Epidermolytic hyperkeratosis	Perinuclear vacuolization of keratinocytes in the upper epidermis, irregular keratohyaline granules, and compact hyperkeratosis

Focal acantholytic dyskeratosis	Acantholysis and dyskeratosis at all levels of the epidermis, suprabasalar clefting, hyperkeratosis, and parakeratosis

Hailey-Hailey-like acantholysis	Prominent acantholysis at all epidermal levels with epidermal hyperplasia and often suprabasal clefting

**Table 2:** Conditions reported in association with described incidental reaction patterns.

Epidermal hyperkeratosis	Acanthoma [1], acrosyringeal epidermolytic papulosis neviformis [2], actinic keratosis [1, 3], atypical/dysplastic nevus [4–6]*, basal cell carcinoma [1, 3]*, epidermoid cyst [3], infundibular cyst [7], dilated hair follicle [4], dilated pore [8], drug-induced acne [9], epidermal nevus [1, 3], granuloma annulare [10], hair follicle [1], hidradenoma [3], intraepidermal sweat duct unit [3], junctional/compound melanocytic nevus [1, 4–6], leukoplakia [11, 12], lichen amyloidosis [10], melanoma [1, 13]*, nevus comedonicus [14, 15], normal oral mucosa [16], nummular eczema [3], reactive erythema [1], scar [1, 3], seborrheic keratosis [1, 10], squamous cell carcinoma [10, 17]*, systemic sclerosis [18], tattoo [1], trichilemmal cyst [10]

Focal acantholytic dyskeratosis	Basal cell carcinoma [19], chondrodermatitis nodularis helicis [19], benign nevi [19, 20], bullous lichen planus [*], condyloma [21, 22], lichen simplex chronicus [*], comedone [19], dermatofibroma [19, 23], fibrous papule [24], hemorrhoids [25, 26], malignant melanoma [19, 20, 27]*, melanocytic nevi with architectural disorder, scars, ruptured follicle, seborrheic keratoses [4, 19, 20, 22], pityriasis rosea [28], pityriasis rubra pilaris [29–31], psoriasis [24, 28], scar [20], squamous cell carcinoma [17], trichofolliculoma [32, 33], vascular nevi/cutis marmorata telangiectasis congenita, vascular twin nevi [34]

Hailey-Hailey-like acantholysis	Acral arteriovenous hemangioma, psoriasis, regressing keratoacanthoma, [35], condyloma acuminatum [28], actinic keratosis [*], basal cell carcinoma [*], benign tumors, malignant tumors [2], seborrheic keratosis [*]

*Current report.
